# Experimental Insights on the Use of Secukinumab and Magnolol in Acute Respiratory Diseases in Mice

**DOI:** 10.3390/biomedicines12071538

**Published:** 2024-07-11

**Authors:** Andrei Gheorghe Vicovan, Diana Cezarina Petrescu, Daniela Constantinescu, Elena Iftimi, Irina Teodora Cernescu, Codrina Mihaela Ancuta, Cezar-Cătălin Caratașu, Laurențiu Șorodoc, Alexandr Ceasovschih, Carmen Solcan, Cristina Mihaela Ghiciuc

**Affiliations:** 1 Department of Morpho-Functional Sciences II—Pharmacology and Clinical Pharmacology, Faculty of Medicine, Grigore T. Popa University of Medicine and Pharmacy of Iași, 16 Universitatii Street, 700115 Iași, Romania; andrei-gheorghe.vicovan@umfiasi.ro (A.G.V.); teodora.cernescu@umfiasi.ro (I.T.C.); cristina.ghiciuc@umfiasi.ro (C.M.G.); 2 Department of Immunology, Faculty of Medicine, Grigore T. Popa University of Medicine and Pharmacy, 16 Universitatii Street, 700115 Iași, Romania; d.constantinescu@umfiasi.ro (D.C.); elena_iftimi@d.umfiasi.ro (E.I.); 3 2nd Rheumatology Department, Clinical Rehabilitation Hospital, 14 Pantelimon Halipa Str., 700664 Iași, Romania; codrina_ancuta@yahoo.com; 4 Rheumatology Department, University of Medicine and Pharmacy “Grigore T Popa”, 16 Universitatii Street, 700115 Iași, Romania; 5 Advanced Research and Development Center for Experimental Medicine (CEMEX), Grigore T. Popa University of Medicine and Pharmacy of Iași, 16 Universității Street, 700115 Iași, Romania; caratasu.catalin@umfiasi.ro; 6 Department of Internal Medicine, Faculty of Medicine, University of Medicine and Pharmacy Grigore T. Popa, 16 Universitatii Street, 700115 Iași, Romania; laurentiu.sorodoc@umfiasi.ro (L.Ș.); alexandr.ceasovschih@umfiasi.ro (A.C.); 7 Department IX—Discipline of Histology, Embryology and Molecular Biology, Faculty of Veterinary Medicine, “Ion Ionescu de la Brad” University of Life Sciences, 3 Mihail Sadoveanu Str., 700490 Iași, Romania; carmensolcan@yahoo.com; 8 Pediatric Emergency Hospital Sf Maria, 700887 Iași, Romania

**Keywords:** acute lung injury, asthma, secukinumab, magnolol, precision medicine, allergic diseases

## Abstract

This study investigates the combined treatment of secukinumab (SECU) and magnolol (MAGN) in a mouse model of LPS-induced ALI overlapped with allergic pulmonary inflammation, aiming to better understand the mechanism behind this pathology and to assess the therapeutic potential of this novel approach in addressing the severity of ALI. The combined treatment reveals intricate immunomodulatory effects. Both treatments inhibit IL-17 and promote M2 macrophage polarization, which enhances anti-inflammatory cytokine production such as IL-4, IL-5, IL-10, and IL-13, crucial for lung repair and inflammation resolution. However, the combination treatment exacerbates allergic responses and increases OVA-specific IgE, potentially worsening ALI outcomes. MAGN pretreatment alone demonstrates higher potency in reducing neutrophils and enhancing IFN-γ, suggesting its potential in mitigating severe asthma symptoms and modulating immune responses. The study highlights the need for careful consideration in therapeutic applications due to the combination treatment’s inability to reduce IL-6 and its potential to exacerbate allergic inflammation. Elevated IL-6 levels correlate with worsened oxygenation and increased mortality in ALI patients, underscoring its critical role in disease severity. These findings offer valuable insights for the advancement of precision medicine within the realm of respiratory illnesses, emphasizing the importance of tailored therapeutic strategies.

## 1. Introduction

Up to 4% of all hospital admissions and more than 10% of all intensive care unit (ICU) admissions are acute lung injury (ALI) and/or acute respiratory distress syndrome (ARDS) patients [1], with a mortality rate of up to 40–70% despite the improvements in intensive care [2], and with evidence of adversely affected long-term quality of life for patients who survive the acute phase of ALI [1]. ALI is characterized by death or dysfunction of alveolar epithelial cells and/or pulmonary capillary endothelial cells, which leads to disruption of the alveolar–capillary barrier [3], while ARDS represents the clinical manifestation of diffuse, severe ALI with increased pulmonary vascular permeability, massive inflammation, and subsequent pulmonary edema and refractory hypoxia [4,5]. Other authors consider ALI as a moderate or mild form of ARDS on a progressive scale [6]. Moreover, there are indirect causative relations between asthma and ALI/ARDS which may also have practical implications on the clinical circumstances in view of the fact that pneumonia, along with aspiration pneumonitis and sepsis, is among the leading causes of ARDS [7].

Early stages of ALI in humans are characterized by elevated interleukin-17A (IL-17A) in circulation and in BALF, and neutrophils have an important role in recruitment, alveolar permeability, and organ dysfunction in ARDS [8,9]. The Th17/Treg imbalance favoring a Th17 shift represents a potential risk indicator in patients with early ARDS due to its association with more pronounced organ dysfunction and worse oxygenation [10]. In a mouse model of LPS-induced ALI, IL-17 aggravated lung inflammation and neutrophil infiltration [11]. On the other hand, inhibition of IL-17 signaling pharmacologically or through genetical modification showed protective effects against development of experimental lung injury [12].

Secukinumab (SECU) is a fully human anti-IL-17 monoclonal antibody approved for treatment of moderate to severe plaque psoriasis, psoriatic arthritis, and ankylosing spondylitis for its ability to selectively neutralize IL-17A. SECU showed modulatory immune effects in various rodent experimental models [13,14,15,16,17,18] and, in high doses, exerts protective effects against severe sepsis-induced ALI in rat models [19]. Beneficial effects of anti-Il-17 therapy in ALI inflammation [20,21] and in alleviation of LPS-exacerbated asthma in murine models [22,23,24,25] were also experimentally proven.

**Figure 1:**
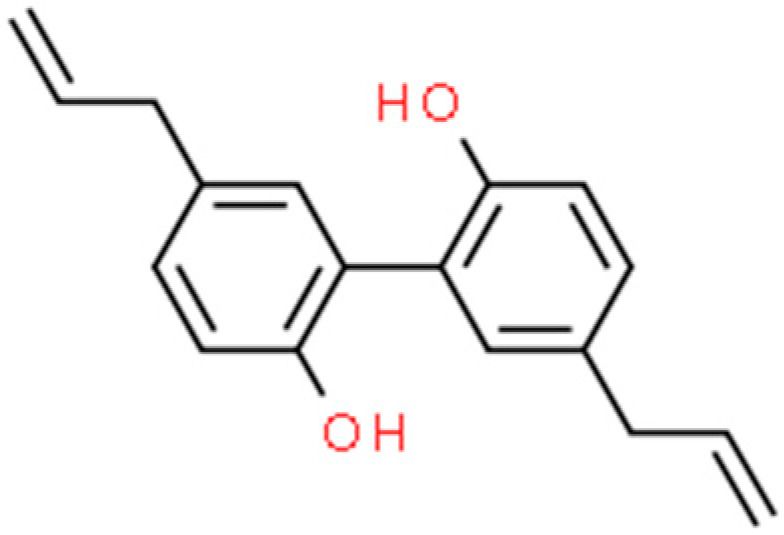
Magnolol chemical structure (https://www.chemspider.com/Chemical-Structure.65251.html, accessed on 21 May 2024).

Magnolol (MAGN), a bioactive ingredient of Magnolia officinalis—Figure 1—is known for anti-inflammatory activity and for the ability to ameliorate endotoxin-induced multiple tissue damage and dysfunction [26,27], including the attenuation of pro-inflammatory cytokines in rodent models of LPS-induced ALI [28,29,30,31]. Among the numerous studies concerning ALI/ARDS treatment, MAGN pretreatment has been noted for its ability to significantly improve severe lung damage (caused by lung edema, alveolar wall thickening, and neutrophil infiltration), to decrease the number of total leukocytes in BALF, and to diminish lung myeloperoxidase (MPO) activity in the lungs of rodents with LPS-induced ALI [28,29,30].

Endotoxins or LPS are derived from the cell wall of gram-negative bacteria and are able to induce a sepsis syndrome associated with key features of ALI: lung recruitment of inflammatory cells, increases in capillary permeability, and subsequent alveolar edema [32]. Previous authors have shown that experimental asthma with intratracheal OVA challenge of earlier OVA-sensitized mice notably exacerbated lipopolysaccharides (LPS)-induced infiltration of leukocytes in bronchoalveolar lavage fluid (BALF) [33].

The individual capacity of both MAGN and SECU in mitigating LPS-induced ALI has been extensively recorded; however, the underlying mechanisms and the specific types of immune responses involved have been only partially clarified. Furthermore, there exists a research gap in terms of a comparative analysis of these two molecules and their interaction, and considering their respective positive impacts on ALI, it can be presumed that a synergistic effect would result from their combination.

In the current investigation, we aimed to bring more clarity in this direction by using a particular murine model of LPS-induced ALI overlapped on OVA-induced allergic pulmonary inflammation, as this type of experimental ALI showed an increased level of severity [33]. We evaluated the levels of cytokines specific to different types of immune responses, such as Th1, Th2, and Th17, following treatment with SECU and MAGN administered either alone or in combination.

As the effects of SECU treatment alone in comparison to saline vehicle control and dexamethasone treatment were previously investigated [18], the current paper is focusing only on the comparative evaluation of SECU treatment, MAGN treatment, and their association.

## 2. Materials and Methods

### 2.1. Chemicals, Reagents, and Antibodies

The following chemicals and drugs were used: phosphate-buffered saline solution (Sigma-Aldrich Chemie GmbH, Taufkirchen, Germany), saline solution (NaCl 0.9%), ovalbumin (GRADE V, Sigma-Aldrich, St. Louis, MO, USA), aluminum hydroxide (Sigma-Aldrich Chemie GmbH, Schnelldor, Germany), LPS (Escherichia coli O127:B8, Sigma-Aldrich Chemie GmbH, Schnelldorf, Germany), NP-40 lysis buffer (Thermo Fisher Scientific, Lancashire, UK), protease inhibitor cocktail (Promega, Madison, WI, USA), ketamine hydrochloride (Ketavet^®^ 100 mg/mL Solution for Injection, Zoetis UK Limited, London, UK), and xylazine hydrochloride (Sigma-Aldrich Chemie GmbH, Schnelldorf, Germany).

Magnolol (98% purity, C_18_H_18_O_2_; M_r_ = 266.3) was purchased from New Natural Biotechnology (Shanghai, China). The CMC-Na (carboxymethylcellulose sodium salt, high viscosity, 700–1500 mPa.s; degree of substitution 0.60–0.95) was procured by Fluka BioChemika (Buchs, Switzerland) and polisorbat 80 (Tween^®^ 80), reagent grade (lot: 19L0956375) was supplied by VWR Chemicals (Solon, OH, USA). All reagents were accompanied by quality certificates. The distilled water was obtained in the private laboratories with a GFL type 2004, no. 11918315J distiller (Germany). All reagents were used without further purification.

Antibodies used treatment and sample processing: anti-IL-17 monoclonal antibody (Secukinumab, Cosentyx^®^, Novartis Pharma GmbH, Nuremberg, Germany), LEGEND MAX™ Mouse OVA Specific IgE ELISA Kit (BioLegend, San Diego, CA, USA), and Luminex Mouse Discovery Assay 8-Plex- IFNgamma, IL-4, IL-5, IL-6, IL-13, IL-17/IL17A, TNF-alpha, VEGF (R&D Systems, Minneapolis, MN, USA).

### 2.2. Preparation Protocol: 2% CMC-Na Mucilage and Magnolol Suspension

Two grams of CMC-Na powder were added in small quantities under stirring in 80 g of water heated to 65 °C, and stirring continued for 15 min until powder was completely dispersed in a water bath mortar. Up to 100 g was made with distilled water, homogenizing gently, avoiding air incorporation. Subsequently, it was allowed to stand for 24 h at room temperature for a complete hydration and structuring process, after 100 g of 2% CMC-Na translucent mucilage was obtained.

The MAGN suspension pharmaceutical formulation was made by dispersing magnolol 0.5% (5 mg/g) in diluted CMC-Na 1% mucilage (obtained by dilution with distilled water). The active substance was gradually dispersed in a small amount of 1% CMC-Na mucilage in the mortar using a pestle, and then 0.25% Tween^®^ 80 was added, stirred, and finally the last portion of mucilage was added by gentle homogenization for 5 min. The suspension for oral use was packaged in sterile plastomer containers and stored in the refrigerator for up to 10 days.

### 2.3. Animal Experimental Design

The Research Ethics Committee of the “Grigore T. Popa” University of Medicine and Pharmacy of Iaşi, Romania approved the study design and protocols (approval No. 332/17.09.2023).

In accordance with Research Law No. 206/27.05.2004 (published by the National Council for Ethics of scientifical Research, Technological Development and Innovation 12.11.2020), 30 female BALB/c mice (17–23 g) obtained from “Cantacuzino” Institute Bucharest were handled and housed in the CEMEX facility of our university in sterile cages under regulated conditions of temperature (22 °C ± 3 °C), humidity (55 ± 5%), and 12 h/12 h day/night cycle. The mice were provided with adequate food and drinking water through the entire period of study. After OVA challenge, female mice develop a more pronounced type of allergic airway inflammation than male mice [34], and therefore only female mice were used for the study.

The animals were divided into four groups (n = 6 in each group):

- OVA + LPS group (positive disease control group)—exposed to ovalbumin inhalation and LPS instillation;
- OVA + LPS + SECU group—exposed to ovalbumin inhalation, LPS instillation, and treatment with secukinumab subcutaneously;
- OVA + LPS + MAGN group—exposed to ovalbumin inhalation, LPS instillation, and pre-/treatment with MAGN by oral gavage;
- OVA + LPS + MAGN + SECU group—exposed to ovalbumin inhalation, LPS instillation, pre-/treatment with MAGN by oral gavage, and secukinumab subcutaneously.

#### 2.3.1. Sensitization Protocol

**Figure 2:**
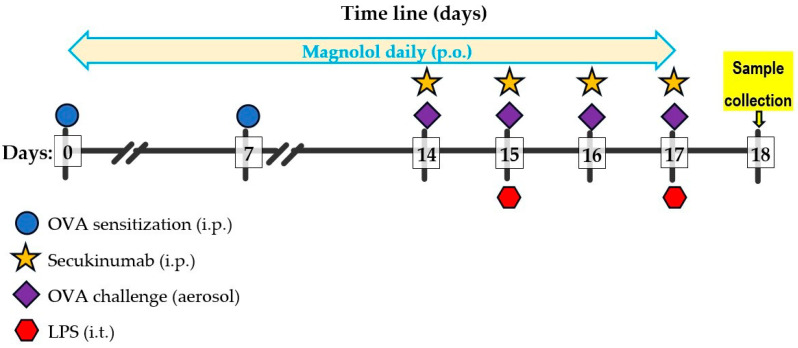
The procedures’ timeline scheme.

Mice underwent a period of acclimatization to the internal environment for seven days before the onset of allergic asthma. The OVA grade V was solubilized to a final concentration of 20 μg/mL in 500 μL of sterile PBS per mouse, with the addition of aluminum hydroxide (alum) to a concentration of 2 mg/mL, following the protocol outlined by Debeuf et al. [35]. The resulting mixture was subjected to rotation for 30 min at room temperature using an end-over-end rotator. The mice belonging to the OVA + LPS, OVA + LPS + MAGN, OVA + LPS + SECU, and OVA + LPS + MAGN + SECU experimental groups were intraperitoneally injected with the OVA/alum solution at a total volume of 0.2 mL per mouse on days 0 and 7, as indicated by prior research [35]. Subsequently, on days 14, 15, 16, and 17, the animals underwent a 25 min challenge in an inhalation chamber with OVA by aerosolizing 10 mL of 1% OVA diluted in PBS for the OVA + LPS, OVA + LPS + MAGN, OVA + LPS + SECU, and OVA + LPS + MAGN + SECU groups, following the methodology outlined by Debeuf et al. [35] (Figure 2).

#### 2.3.2. ALI Induction and SECU Treatment

On days 15 and 18, the intratracheal LPS administration was performed 1 h after OVA aerosol under general anesthesia with ketamine [80 mg/kg mouse bodyweight (BW)] and xylazine (10 mg/kg BW) intraperitoneally, as described in the protocol of Ehrentraut et al. [36]. On day 18, all animals were euthanized in deep anesthesia (ketamine and xylazine overdose) by atlanto-occipital dislocation.

#### 2.3.3. Drug Administration

MAGN at 50 mg/kg body weight was administered to the OVA + LPS + MAGN and OVA + LPS + MAGN + SECU groups daily via oral gavage, from the first day of OVA exposure (day 0) to day 18, as described by Huang et al. [37]. On the days of OVA challenge or SECU treatment, mice received MAGN 60 min prior to each aerosol challenge or SECU injection, respectively.

On days 14, 15, 16, and 17, SECU was administrated at a dose of 10 mg/kg subcutaneously 1 h prior to each OVA aerosol [17]. The doses of SECU were established from similar experiments on mice [17,38].

#### 2.3.4. Sample Collection of Blood for OVA-Specific IgE

Mice were exsanguinated from the vena inguinalis immediately after loss of pedal withdrawal reflex (i.e., no response to a toe pinch). The blood was collected in 0.5 mL microcentrifuge tubes and the serum separated from whole blood by centrifugation (10 min; 3000 revolutions per min; 4 °C) for determination of OVA-specific IgE using a Legend Max Mouse OVA Specific IgE ELISA kit (Biolegend, Sandiego, CA, USA), an Infinite 200 PRO M Plex Tecan plate reader (Tecan, Grodig, Austria), and Magellan v 7.4 software (Tecan, Grodig, Austria).

#### 2.3.5. Sample Collection of BALF for Cytokines

BALF was obtained via tracheal cannulation using three consecutive infusions/aspirations of ice-cold PBS (0.5 mL each), totaling 1.5 mL, followed by centrifugation at 250× *g* for 5 min at 4 °C. The resultant supernatant was then transferred to a new Eppendorf tube, centrifuged at 10,000× *g* for 15 min, divided into 150 µL aliquots, and preserved at −80 °C until cytokine assessment (RD-LXSAMSM-08 Luminex Mouse Discovery Assay 8-Plex: IFN-γ, IL-4, IL-5, IL-6, IL-13, IL-17/IL17A, TNF-alpha, VEGF). Following vortex mixing, 5 µL from the remaining cell pellet was utilized for slide preparation for differential counting, and subsequently stained with May–Grünwald (Sigma-Aldrich, 1.01424) and Giemsa (Sigma-Aldrich, 1.09204). The average cell count per microscopic field was calculated using an optical microscope with a dry objective lens (400× total magnification) by examining 10 fields. The identification of neutrophils, eosinophils, lymphocytes, and macrophages was carried out using an optical microscope with an immersion objective lens (1000× total magnification) by analyzing 200 cells.

#### 2.3.6. Analysis of Cytokines in Lung Homogenate

The right pulmonary lobes were excised subsequent to the retrieval of bronchoalveolar lavage fluid, assessed for mass, and subsequently immersed in 200 µL of NP-40 lysis buffer (Thermo Fisher Scientific, UK) supplemented with protease inhibitor cocktail (Promega, USA) and placed on ice for a duration of 15 min. Subsequent to that, manual grinding was carried out on ice utilizing a 1 mL Wheaton Tenbroeck tissue grinder. The resulting homogenized mixture underwent centrifugation at 10,000× *g* for 15 min, followed by preservation of 60 µL portions of the resulting supernatant at −80 °C until further analysis.

The measurement of cytokine levels in lung tissue extracts for IFN-γ, IL-4, IL-5, IL-6, IL-13, IL-17/IL17A, TNF-α, and VEGF was carried out utilizing an RD-LXSAMSM-08 Luminex Mouse Discovery Assay 8-Plex (R&D Systems, Minneapolis, MN, USA) and a Gen-Probe Luminex 100/200 xMAP platform (Austin, TX, USA). The specimens were diluted by a factor of 1.25 in RD 6–52 and handled according to the manufacturer’s instructions.

#### 2.3.7. Histological Analysis of Lung Tissue

Following the collection of BALF, the lower lobe of the left lung was excised and then placed in a solution containing 4% formaldehyde for fixation. The tissue was subsequently dehydrated using varying concentrations of ethyl alcohol, followed by clarification with xylene, impregnation, and embedding in paraffin. Sections of the tissue, measuring at 5 μm, were then prepared and stained using Hematoxylin Eosin (H.E.) as well as periodically with Schiff acid (PAS) for further analysis. The histological structures were examined and measured using a Leica microscope, and photomicrographs were taken for documentation and analysis purposes.

### 2.4. Statistical Analysis

The data obtained are presented as the means ± standard error (SE). Figures are depicted in bar formats along with standard error and asterisks denoting significant differences in comparison to the SAL group. In order to assess the statistically significant distinctions among multiple groups, the parametric one-way analysis of variance (ANOVA) was employed, followed by either the Holm–Šidák method for multiple comparisons or the non-parametric Kruskal–Wallis one-way analysis of variance on ranks. All statistical analyses were conducted using the Statistical Package for the Social Sciences (SPSS) version 27.0 (SPSS Inc., Chicago, IL, USA) software. A *p*-value of less than 0.05 was deemed to be statistically significant.

## 3. Results

### 3.1. Effects of SECU, MAGN, and MAGN + SECU Combined Treatment, Respectively, on the Level of Ovalbumin (OVA)-Specific IgE in Serum

**Figure 3:**
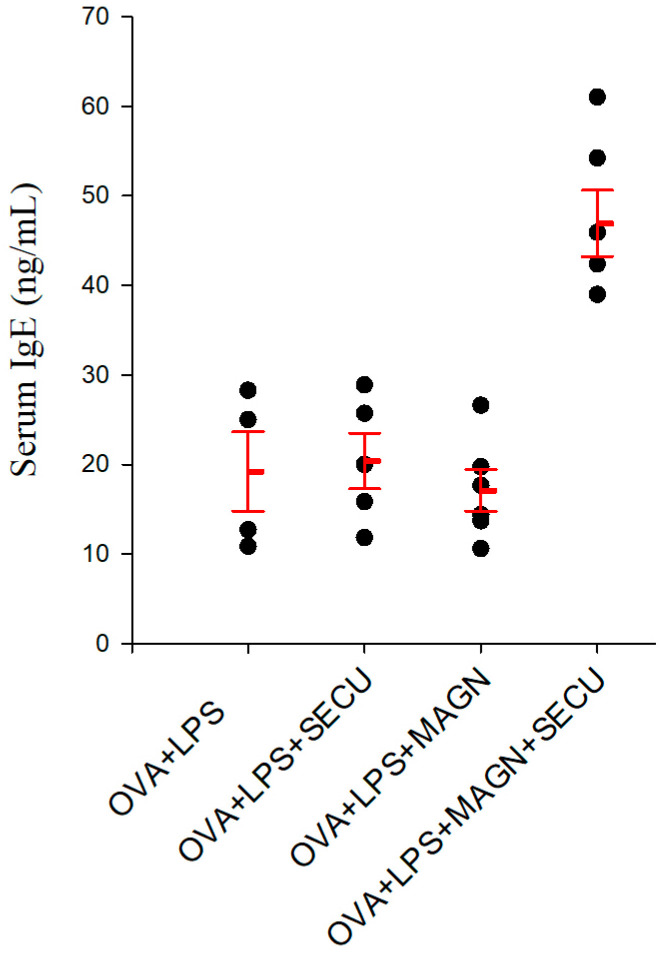
IgE concentration in serum (ng/mL). Values are expressed as mean ± standard error; n = 5. OVA + LPS: positive disease control group; OVA + LPS + MAGN: magnolol-treated group; OVA + LPS + SECU: secukinumab-treated group; OVA + LPS + MAGN + SECU: combined magnolol- and secukinumab-treated group. Red lines: mean ± SE; black dots: individual data.

Figure 3 reveals increased OVA-specific IgE concentration in all OVA-exposed groups, confirming the OVA sensitization. The apparent benefits of MAGN cannot be confirmed due to the lack of statistical significance from the OVA + LPS (positive disease control group) or secukinumab-treated group. Surprisingly, the association of MAGN and SECU treatments seems to determine an increase of OVA-specific IgE serum concentration in comparison to all other OVA-exposed groups.

### 3.2. Effects of SECU, MAGN, and MAGN + SECU Combined Treatment, Respectively, on Bronchoalveolar Lavage Fluid (BALF)

#### 3.2.1. Cell Count in BALF

**Figure 4:**
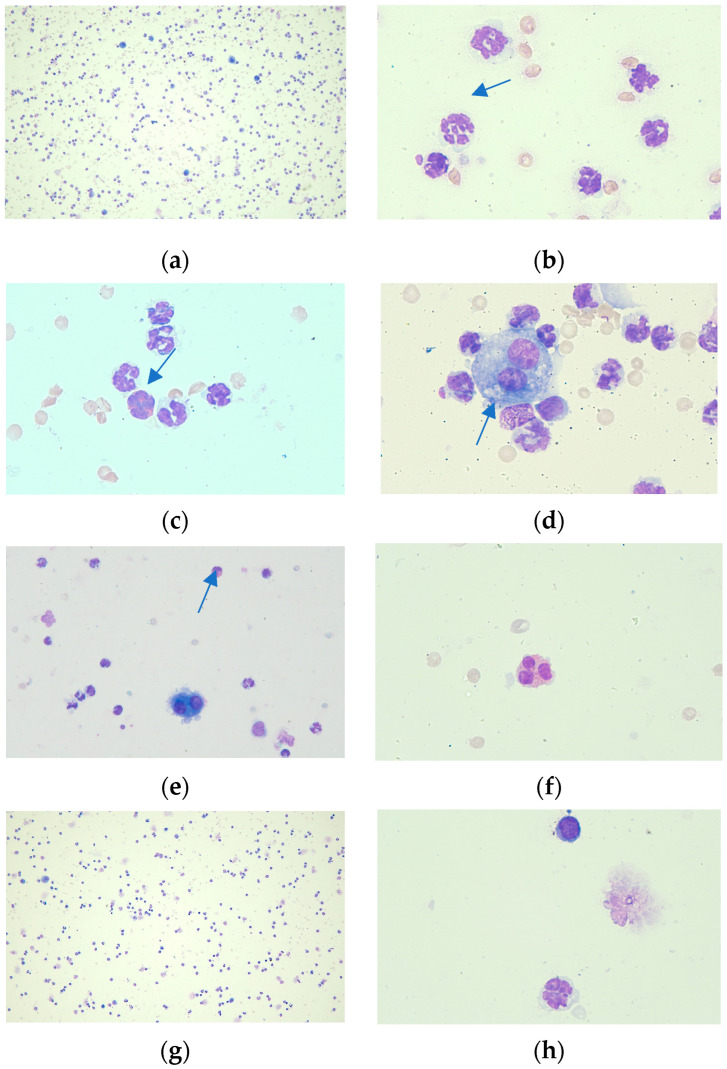
Differential cell count, optical microscopy: (**a**) OVA + LPS group: cellularity, ×10 dry objective; (**b**) OVA + LPS group: neutrophils, hypersegmented neutrophil (arrow), ×100 immersion objective; (**c**) OVA + LPS + SECU group: neutrophils and an eosinophil (arrow), ×100 immersion objective; (**d**) OVA + LPS + SECU group: binucleated alveolar macrophage (arrow) and neutrophils, ×100 immersion objective; (**e**) OVA + LPS + MAGN group: binucleated alveolar macrophage, neutrophils and an eosinophil (arrow), ×40 dry objective; (**f**) OVA + LPS + MAGN group: eosinophil, ×100 immersion objective; (**g**) OVA + LPS + MAGN + SECU group: cellularity, ×10 dry objective; (**h**) OVA + LPS + MAGN + SECU group: neutrophil and lymphocyte, ×100 immersion objective.

In Figure 4, the alterations in inflammatory cells in BALF for macrophages, neutrophils, lymphocytes, and eosinophils are illustrated for each group. The depiction of the eosinophil in picture (f) exhibits remarkable similarities in aspect to the specific subtype of SiglecF^+^Gr1^hi^ eosinophils [39].

**Figure 5:**
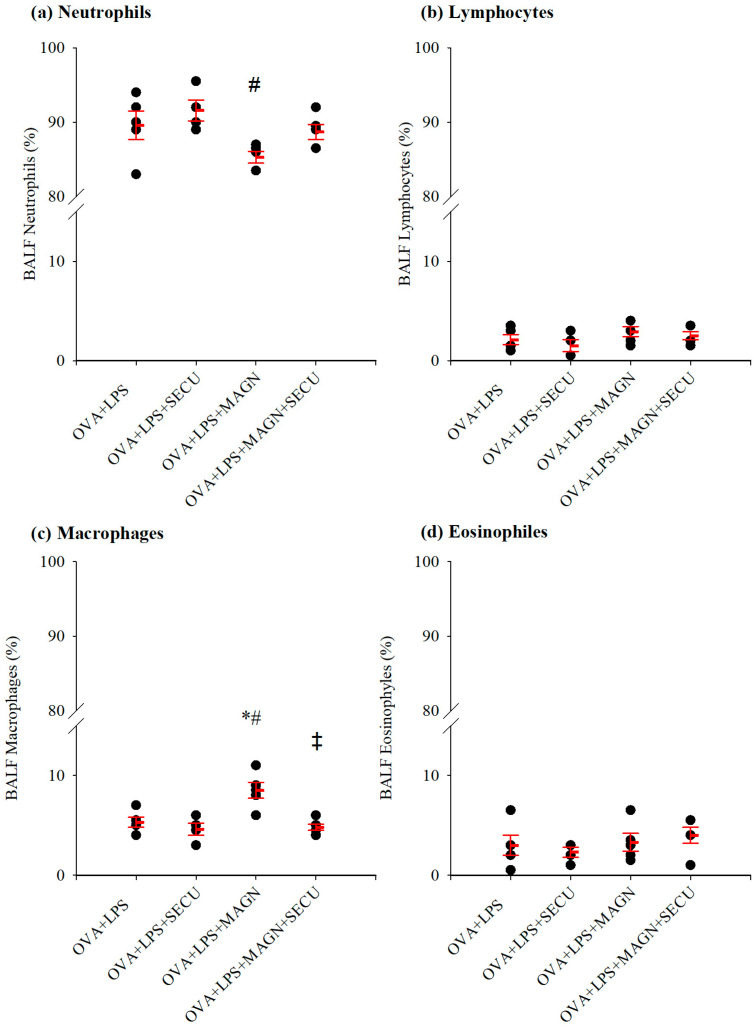
Graphical illustration of the differential cell counts expressed in percentages for (**a**) macrophages, (**b**) neutrophils, (**c**) lymphocytes, and (**d**) eosinophils. Values are expressed as mean ± standard error; n = 5. * *p* < 0.05 vs. OVA + LPS group; # *p* < 0.05 vs. OVA + LPS + SECU group; ‡ *p* < 0.05 vs. OVA + LPS + MAGN + SECU group; as determined by one-way ANOVA, followed by the Holm–Šidák method for multiple comparisons. OVA + LPS: positive disease control group; OVA + LPS + MAGN: magnolol-treated group; OVA + LPS + SECU: secukinumab-treated group; OVA + LPS + MAGN + SECU: combined magnolol- and secukinumab-treated group. Red lines: mean ± SE; black dots: individual data.

Percentual quantification in BALF of differential cell counts for neutrophils, lymphocytes, macrophages, and eosinophiles reveals a significant decrease of neutrophils in the case of MAGN treatment solely (*p* < 0.05). On the other hand, MAGN showed the potential to slow down the decline of macrophages comparative to SECU treatment alone or to the positive disease control group (*p* < 0.05). The tendency of SECU to decrease macrophages can also be observed in the MAGN and SECU combined treatment (Figure 5).

There were no statistically significant differences among the counts of lymphocytes and eosinophils in any of the groups.

#### 3.2.2. Cytokines in BALF

**Figure 6:**
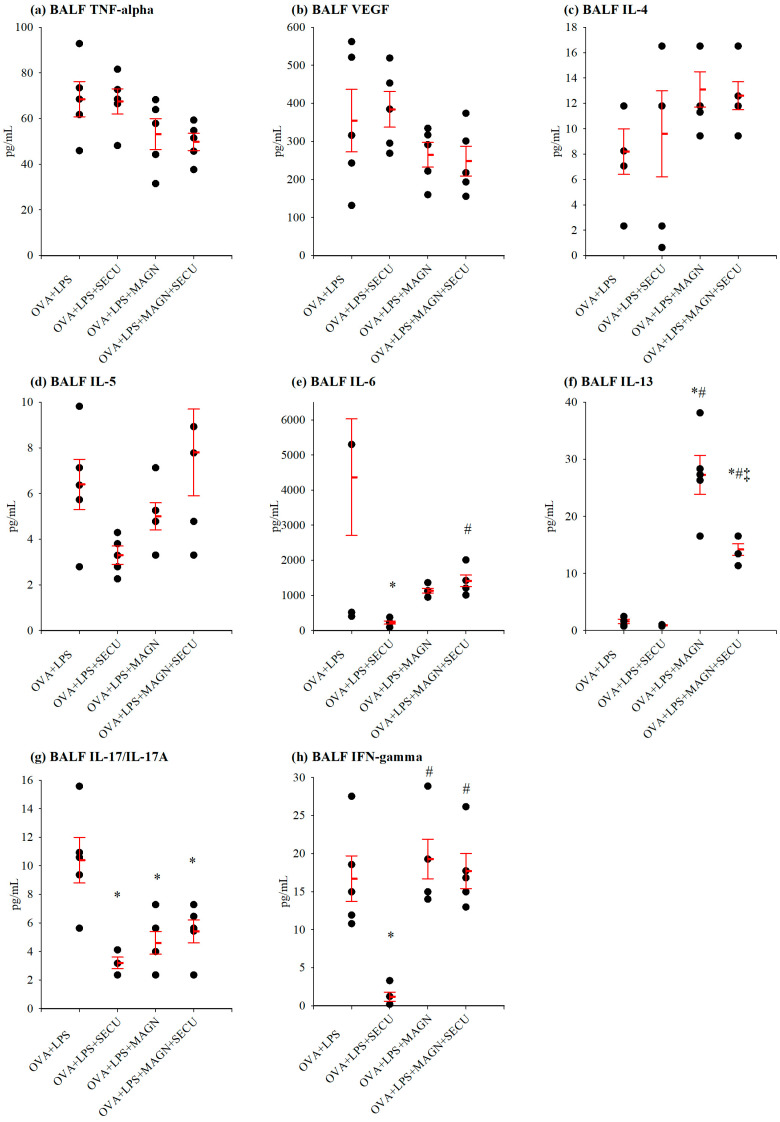
Cytokine concentration (pg/mL) in BALF: (**a**) TNF-α, (**b**) VEGF, (**c**) IL-4, (**d**) IL-5, (**e**) IL-6, (**f**) IL-13, (**g**) IL-17, and (**h**) IFN-γ. Values are expressed as mean ± standard error; n = 5. * *p* < 0.05 vs. OVA + LPS group; # *p* < 0.05 vs. OVA + LPS + SECU group; ‡ *p* < 0.05 vs. OVA + LPS + MAGN + SECU group; as determined by one-way ANOVA, followed by the Holm–Šidák method for multiple comparisons. OVA + LPS: positive disease control group; OVA + LPS + MAGN: magnolol-treated group; OVA + LPS + SECU: secukinumab-treated group; OVA + LPS + MAGN + SECU: combined magnolol- and secukinumab-treated group. Red lines: mean ± SE; black dots: individual data.

Representative cytokines for specific Th1, Th2, and Th17 immune responses were evaluated in BALF for each investigated group (Figure 6).

SECU treatment alone has clearly shown the ability to decrease the level of IL-6 in BALF. The association of MAGN pretreatment to SECU was not able to potentiate this effect on IL-6, but surprisingly did the opposite in the case of Il-13: SECU treatment paired with MAGN pretreatment had an inhibitory role on IL-13 increase in comparison to administration solely of MAGN that undoubtedly induced an increment of IL-13 in BALF.

Both SECU and MAGN, whether used alone or in combination, resulted in a reduction of IL-17. Unfortunately, the distinctions in their impacts do not reach statistical significance, thus making it challenging to determine comparatively whether any of them or their combination exerts a superior impact on IL-17.

Interestingly, while SECU treatment decisively decreases the level of INF-γ, MAGN pretreatment acts as a strong stimulus for IFN-γ increase. Even when it is administrated in addition to SECU, the stimulatory influence of MAGN categorically counteracts the inhibitory effect of SECU on IFN-γ.

In addition to the previously cited cytokines, the quantity of VEGF was assessed in BALF for each group. There was no notable alteration observed as a result of any of the treatments studied or their amalgamation (Table 1).

#### 3.2.3. Cytokines in Lung Tissue Homogenate (LTH)

**Figure 7:**
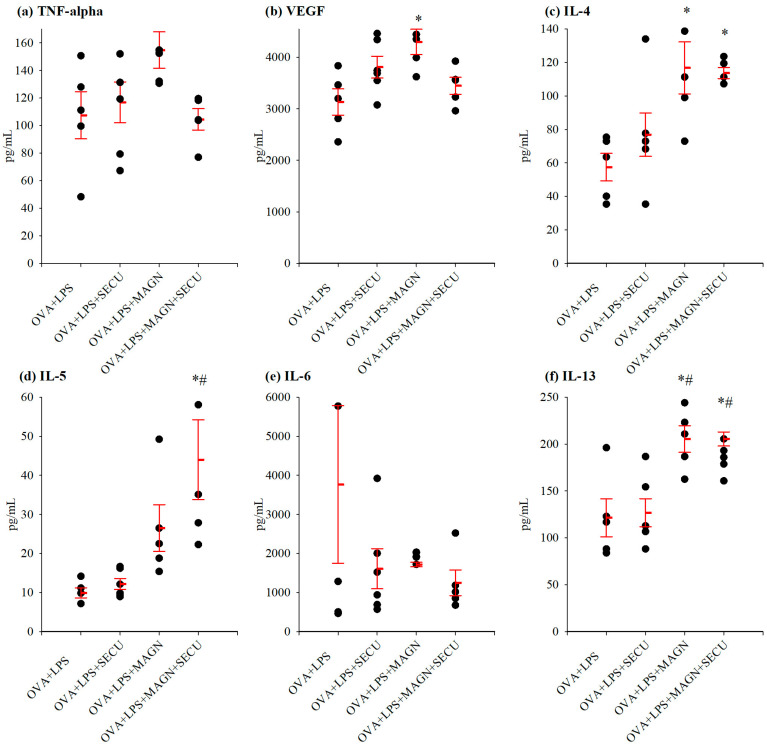
Cytokine concentration (pg/mL) in lung tissue homogenate: (**a**) TNF-α, (**b**) VEGF, (**c**) IL-4, (**d**) IL-5, (**e**) IL-6, (**f**) IL-13, (**g**) IL-17, and (**h**) IFN-γ. Values are expressed as mean ± standard error; n = 6. * *p* < 0.05 vs. OVA + LPS group; # *p* < 0.05 vs. OVA + LPS + SECU group; as determined by one-way ANOVA, followed by the Holm–Šidák method for multiple comparisons. OVA + LPS: control group—positive for disease; OVA + LPS + MAGN: magnolol-treated group; OVA + LPS + SECU: secukinumab-treated group; OVA + LPS + MAGN + SECU: combined magnolol- and secukinumab-treated group. Red lines: mean ± SE; black dots: individual data.

To acquire a comprehensive understanding of the interaction among various immune reactions, the identical cytokines evaluated in BALF were measured in LTH (Figure 7).

VEGF evaluation in LTH revealed an increase under MAGN pretreatment compared to the positive disease control group. Unfortunately, the significance of this rise cannot be thoroughly and comparatively appraised due to statistically questionable data concerning the impact of SECU therapy alone on VEGF dynamics.

MAGN has demonstrated a highly efficacious stimulatory impact on IL-4 in this particular pathological induced setting, as both in monotherapy and combined with SECU therapy it led to a clearly discernible elevation of this cytokine.

Regarding the influence on IL-5, only the increase under combined treatment can be considered, as this is the only one measured as statistically noteworthy. However, the substantial rise appears to be predominantly attributed to MAGN.

Since IL-13 level is significantly raised in both the OVA + LPS + MAGN and OVA + LPS + MAGN + SECU groups in comparison to the positive disease control group, the enhancing impact can appropriately be ascribed to MAGN.

The impact of MAGN on the concentration of IFN-γ is undeniably characterized by a stimulating effect when compared to SECU. The robustness and potency of this influence are further underscored and substantiated by the fact that it continues to manifest itself even in the case of SECU co-administration.

#### 3.2.4. Histological Analysis

**Figure 8:**
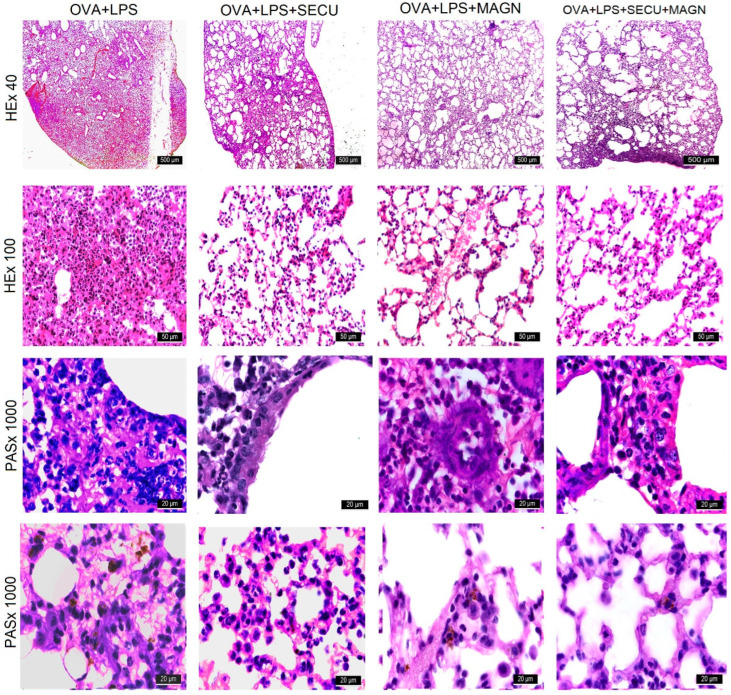
Representative HE- and PAS-stained tissue sections of lungs and bronchia from mice in ovalbumin-induced asthma exacerbated with LPS administration. Original magnification for HE: ×40 or ×100, for PAS: ×1000. SAL: normal control group; OVA + LPS: positive disease control group; OVA + LPS + MAGN: magnolol-treated group; OVA + LPS + SECU: secukinumab-treated group; OVA + LPS + MAGN + SECU: combined magnolol- and secukinumab-treated group.

The lung histological alterations induced by the presence of OVA + LPS involve an augmentation in the size and quantity of goblet cells in the pseudostratified ciliated epithelium that lines the airways. This epithelial layer exhibits disruptions in the connections between its cells, which are discernible under light microscopy. Additionally, there is edema around blood vessels and bronchial tubes, along with an infiltration of neutrophilic polymorphonuclear cells, eosinophils, lymphocytes, and peribronchial macrophages. Within the bronchial lumen, polymorphonuclear cells are visible. Alveoli in the lungs are collapsed, with an increase in the thickness of the septal walls. Neutrophils, monocytes, eosinophils, alveolar macrophages, and lymphocytes can be identified within both the septal walls and the alveolar lumens (Figure 8 and Table 1).

In the context of SECU treatment only, a notable reduction is observed in the quantity of neutrophils, persisting at diminished levels in the vicinity of the bronchi. The pulmonary alveoli exhibit a diminished thickness in their walls and a decreased presence of figurative elements within the lumen or septal structures, and instances of alveolar collapse are infrequent.

The OVA + LPS + MAGN group demonstrates a compact, reduced region in contrast to the OVA + LPS + SECU group, within the central zone of the lobules, where the pulmonary alveoli are compressed and predominantly invaded by neutrophils, lymphocytes, and macrophages.

The aspect of the OVA + LPS + SECU + MAGN group unveils even less pronounced pathological modifications compared to the OVA + LPS + MAGN group.

There is a consistent decrease of neutrophils, leading to a significant reduction in their presence, particularly around the bronchi. Furthermore, an observable characteristic of the lung alveoli within this group is the presence of a thinner wall, along with an obviously diminished count of figurative elements found either in the lumen or the septal wall. Additionally, the collapsing of the lung alveoli is a rare occurrence in this group.

The level of lung inflammation and caliciform cell hyperplasia was assessed subjectively on a scale ranging from 0 to 4: 75%. In order to quantify caliciform cells in bronchi and bronchioles, a five-point classification system was utilized, with categories including 0: <0.5% PAS-positive cells and 1: <25%, 2: 25–50%, 3: 50–75%, and 4: >75%. Each slide was evaluated by counting five fields, and the mean score was derived from observations of five animals. The quantification of PAS-positive caliciform cells was determined as the number of positive PAS cells per millimeter of basement membrane to adjust for the size of the airway [40]. To mark inflammatory cell infiltration in the intraluminal, alveolar, peribronchial, and perivascular regions, cell counting was performed blindly based on a five-point grading system for the following characteristics: 0: normal, 1: few cells, 2: a ring of inflammatory cells, a deep cell layer; 3: a ring of inflammatory cells 2–4 cells deep, 4: a ring of inflammatory cells >4 cells deep [40].

## 4. Discussion

The current research assesses the immunomodulatory impacts of combined treatment with SECU and MAGN on some of the specific cytokines associated with the Th1, Th2, and Th17 axes of the immune response in a murine experimental model of LPS-induced ALI overlaid on OVA-induced allergic pulmonary inflammation. We choose this experimental model of particular degree of severity based on the apparent deleterious influence of preexisting allergic asthma on ALI outcome [33]. The results of SECU treatment alone in comparison to the SAL (saline) and OVA + LPS + DEXA group (control group—positive for treatment) were previously published [18].

The increased OVA-specific IgE serum concentration under MAGN and SECU combined treatment, when contrasted with the levels in all the other groups exposed to OVA, appears to have a detrimental effect within the framework of ALI.

The effects of SECU and MAGN combined use on ALI at the level of lung tissue histology might be interpreted as a “moment picture” of the dynamic interplay among different branches of the immune system (Th1, Th2 or Th17), rather than as a definitive end outcome.

In the specific design of our ALI experimental model, the Th2 immune response is significantly overshadowed by the strength of the non-Th2 immune response triggered by the high-exposure dose of LPS. This immune response shift can be partly explained by the ability of IFN-γ to inhibit the baseline state of Th2 activation in subjects with asthma by mechanisms involving the inhibitory effect of type 1 cytokines on Th2 inflammation [41].

This predominance of non-Th2 response is contrasted with the increased levels of IgE. One possible reasoning could be the marked increase in IL-13, which, independently of IL-4, stimulates the maturation of B cells and significantly promotes IgE synthesis [42,43].

The tissular damage observed in the untreated group results primarily from the infiltration of neutrophils induced by the Th17 response. However, the mitigation of ALI effects at the level of lung tissue histology under combined use of SECU with MAGN has two possible components: IL-17 blockade by SECU and inhibitory effects of MAGN on neutrophils activity [44,45]

MAGN pretreatment showed higher potency on neutrophil decrease than SECU in our experimental model of increased degree of severity of ALI given the preexistence of asthma. Neutrophils play a pivotal role in acute lung inflammation, primarily through their involvement in the pathogenesis of conditions such as ALI and acute respiratory distress syndrome (ARDS). Our results may indicate a favorable influence of MAGN pretreatment in relation to the degree of asthma severity. The utilization of MAGN pretreatment might ameliorate the symptoms experienced by individuals suffering from asthma. Other authors found that pretreatment i.p. with MAGN (5, 10 and 20 mg/kg i.p.) significantly decreased the number of neutrophils (*p* < 0.01) in BALF [29] and that SECU exerted beneficial effects in ALI inflammation [20,21]. Upon activation, neutrophils migrate to the lungs, where they release a variety of pro-inflammatory mediators, including reactive oxygen species (ROS), cytokines, and proteolytic enzymes, which contribute to tissue damage and inflammation. Neutrophils also form neutrophil extracellular traps (NETs), which, while essential for trapping pathogens, contribute to ALI pathogenesis by releasing pro-inflammatory and cytotoxic molecules that exacerbate thromboinflammation and lung tissue injury [46]. Additionally, IL-17 has been shown to enhance neutrophil functions, including ROS production and NET formation, thereby amplifying lung tissue damage during inflammation [47]. Despite their critical role in host defense, the dysregulated and prolonged presence of neutrophils in the lungs can lead to severe tissue damage and impaired resolution of inflammation, highlighting the need for therapeutic strategies that can modulate neutrophil activity without compromising their protective functions.

Our study results revealed the presence of SiglecF^+^Gr1^hi^ eosinophils in BALF of the MAGN-treated group in optical microscopy (Figure 4—picture (f)). These eosinophils represent a distinct subpopulation within the lungs of allergen-challenged mice, characterized by a unique cytokine profile that includes lymphocyte-targeting cytokines, suggesting their pivotal role in modulating immune responses during allergic inflammation [39]. These eosinophils express IL-13 at significant levels (72 pg/10^6^ cells) [39], indicating their potential role in enhancing transition from a pro-inflammatory to an anti-inflammatory macrophage phenotype. Alternative macrophage activation is induced by type 2 cytokines, IL-4 and IL-13 [48,49]. In the early stages of ARDS, classically activated (M1) macrophages dominate, secreting pro-inflammatory cytokines to clear pathogens, which may inadvertently damage alveolar epithelial cells and contribute to cell death. As the condition progresses, the presence of alternatively activated macrophages (M2s) becomes more pronounced. These M2 macrophages secrete anti-inflammatory cytokines that dampen the inflammatory response, thereby promoting epithelial regeneration and alveolar structure remodeling, crucial for the recovery phase of ARDS [50]. Their ability to switch from a pro-inflammatory phenotype in the early stages of injury to a pro-repair phenotype is crucial for effective lung repair [51]. The metabolic activity of M2, influenced by immunometabolism, plays a significant role in their function during lung repair. The metabolic intermediates produced by M2s support their polarization and function, highlighting the importance of their differentiation in the regulation of macrophage activity during lung repair [49]. Moreover, M2s are involved in the clearance of pathogens and debris, a process essential for initiating tissue repair [52]. M2s are central to the process of lung repair, contributing to the resolution of inflammation, clearance of debris, and tissue remodeling. This particular context could potentially offer a favorable outlook when interpreting the rise of macrophages following MAGN treatment as advantageous. SiglecF^+^Gr1^hi^ eosinophils also uniquely express lymphocyte-targeting cytokines such as CXCL13 and IL-27, which are involved in the regulation of B and T lymphocyte functions, further indicating their capacity to modulate immune responses in a manner that could exacerbate inflammatory conditions [39]. IL-27 targets Tregs for anti-inflammatory functions in allergic inflammation, and altered IL-27 responsiveness in Tregs may perpetuate inflammation [53]. The administration of IL-27 via intranasal route has been shown to mitigate Th2-mediated allergic lung inflammation and restructuring in murine asthma models through the restoration of both STAT1 and STAT3 signaling pathways [54].

The elevated production of IL-13 is able to suppress Th17 responses by direct inhibition of IL-23, IL-1beta, and IL-6 expression (according to Kleinschek et al., 2007) [55]. Moreover, alternative macrophage activation is induced by type 2 cytokines like IL-13 [48,49]. Alternatively activated macrophages secrete anti-inflammatory cytokines that dampen the inflammatory response, thereby promoting epithelial regeneration and alveolar structure remodeling, crucial for the recovery phase of ARDS [50]. A recent study by Percopo et al. (2016) showed that the elevated level of Il-13 concentration can be primarily linked to SiglecF^+^Gr1^hi^ eosinophils, among various other sources [39]. We assume an increased ratio of this distinct subpopulation of eosinophils as explanation for the high levels of IL-13 in BALF of the MAGN-treated group. However, it remains for further research to confirm by flow cytometry the presence of Gr1 antigen (the marker for SiglecF^+^Gr1^hi^ eosinophils subpopulation) on the surface of eosinophils and, eventually, the quantification of it by the FACS (fluorescence-activated cell sorting) technique. If there is any causal relation between MAGN and the increased presence of SiglecF^+^Gr1^hi^ eosinophils, additional studies must be conducted to ascertain the molecular targets responsible for this presumed phenomenon.

Contrary to expectations, the combination of MAGN pretreatment and SECU was found to be ineffective in reducing IL-6 compared to SECU treatment alone. Moreover, this association decreased SECU potency to diminish IL-6 in BALF. Clinically, the role of IL-6 in ALI is well documented. IL-6’s association with the oxygenation index in ALI patients indicates its role as a predictive biomarker for disease severity, where elevated levels correlate with worsened oxygenation, suggesting a direct impact on the physiological severity of lung injury [56]. Elevated plasma IL-6 levels were also associated with a decreased number of ventilator-free days (VFDs), highlighting its prognostic value in predicting more severe outcomes and longer dependency on mechanical ventilation in ALI patients [56]. The significant association of IL-6 with mortality in a larger cohort within the study underscores its critical role in the pathogenesis of ALI and its potential utility in identifying patients at higher risk of death, thereby guiding more aggressive or targeted therapeutic interventions [56]. In rodent models of sepsis [57] and LPS-induced mastitis [58], MAGN exhibited a dose-dependent anti-inflammatory effect. Moreover, increased levels of TNF-α, IL-1β, and IL-6 in lungs were markedly reduced by magnolol in LPS-induced ALI in rats [30].

The elevated IL-4 in LTH induced by MAGN either in monotherapy or combined with SECU might be an important contributor for the shift of macrophages towards the M2 subtype responsible for the production of IL-13, IL-5, TGFβ, and IL-10 [49]. Only in LTH do our results demonstrate the ability of combined SECU and MAGN treatment to induce a significant increase of IL-5—the cytokine predominantly responsible for differentiation of eosinophils in the anti-inflammatory CD101^−^ phenotype [33].

The combination of SECU and MAGN treatments induced IL-13 increase both in BALF and LTH. Besides alternative macrophage activation, elevated production of IL-13 is required for suppression of Th17 responses by direct inhibition of IL-23, IL-1β, and IL-6 expression in activated dendritic cells [55].

In contrast to SECU, MAGN has the potential to boost IFN-γ in both BALF and LTH even in association with SECU. This effect may be beneficial due to the protective role of IFN-γ in cytokine release syndrome-induced extrapulmonary ALI by modulating immune responses and reducing tissue damage [59].

Both studied treatments, either as monotherapies or combined, exhibited an inhibitory effect on IL-17. This validates their therapeutic potential in ALI even from the early stages characterized by elevated levels of interleukin-17A (IL-17A) in the blood and BALF [8,9] and an imbalance between Th17 and Treg cells, favoring a Th17 shift [10].

It is important to highlight the significant difference observed in the study designs, which, in addition to the varying doses of MAGN and LPS given, included the differing durations for MAGN pretreatment, with a particular focus on the inclusion of asthma as a preexisting condition solely within our study.

- Histological modifications for MGN-treated groups are consistent with those of Ni et al., who found minor histopathological changes in lungs from mice with LPS-induced ALI under MAGN treatment, especially in inflammatory cell infiltration [28].
- Finally, it should be taken into account that biologic therapies targeting IL-17 axis administration in atopic patients may require more frequent switching among biologics to control their active disease compared to non-atopic patients [60].
- Study limitations:
- The resulting values for cytokines in BALF and LTH represent only a status quo at the sampling moment. This restrains our view to a “moment picture” without any information regarding the dynamic evolution;
- Results from murine models can present challenges in translation of findings to the intricate and distinct human disease of asthma. Additionally, utilizing exclusively female animals may prompt inquiries regarding the potential impact on male mice;
- MGN has downsides like reduced bioavailability in rodents—less than 10% for oral administration [61]—or the potential inhibition of UDP-glucuronosyltransferase (UGT) enzyme activity isoforms 1A7 and 1A9 in humans and rodents [62,63]. Inhibition of UGTs by magnolol may be a potential mechanism that enhances the toxicity of drugs or other active compounds contained in the herbal preparation. Because of these disadvantages, future research should consider natural sources for drugs with a higher variability and abundance, like marine drugs [64].

## 5. Conclusions

The combined treatment with SECU and MAGN in ALI models shows a complex immunomodulatory impact, with both beneficial and detrimental effects. It promotes M2 macrophage polarization, enhancing production of anti-inflammatory cytokines such as IL-4, IL-5, IL-10, and IL-13, which are crucial for lung repair and inflammation resolution. Additionally, it inhibits IL-17, reducing early-stage inflammation. MAGN pretreatment alone demonstrates a higher potency in reducing neutrophils and enhancing IFN-γ, suggesting its potential in mitigating severe asthma symptoms and modulating immune responses. On the other hand, while both treatments inhibit IL-17 and promote M2 macrophage polarization, the combination may exacerbate allergic responses and increase OVA-specific IgE, potentially worsening ALI outcomes. Moreover, the combination treatment’s inability to reduce IL-6 and its potential to exacerbate allergic inflammation highlight the need for careful consideration in therapeutic applications. These findings could offer valuable insights for the advancement of precision medicine within the realm of respiratory illnesses.

## Figures and Tables

**Table 1:** Lung inflammation scores for caliciform cell hyperplasia (CCH) and inflammatory cell infiltration (ICI).

Group	OVA + LPS	OVA + LPS + SECU	OVA + LPS + MAGN	OVA + LPS + MAGN + SECU
CCH Score	4	2	2	1
ICI Score	4	3	2	1

## Data Availability

The data presented in this study are available on request from the corresponding author.

## References

[1] Bellani G., Laffey J.G., Pham T., Fan E., Brochard L., Esteban A., Gattinoni L., van Haren F., Larsson A., McAuley D.F. (2016). Epidemiology, Patterns of Care, and Mortality for Patients with Acute Respiratory Distress Syndrome in Intensive Care Units in 50 Countries. JAMA.

[2] Rubenfeld G.D., Caldwell E., Peabody E., Weaver J., Martin D.P., Neff M., Stern E.J., Hudson L.D. (2005). Incidence and outcomes of acute lung injury. N. Engl. J. Med..

[3] Matthay M.A., Zemans R.L., Zimmerman G.A., Arabi Y.M., Beitler J.R., Mercat A., Herridge M., Randolph A.G., Calfee C.S. (2019). Acute respiratory distress syndrome. Nat. Rev. Dis. Primers.

[4] Fan E., Del Sorbo L., Goligher E.C., Hodgson C.L., Munshi L., Walkey A.J., Adhikari N.K.J., Amato M.B.P., Branson R., Brower R.G. (2017). An Official American Thoracic Society/European Society of Intensive Care Medicine/Society of Critical Care Medicine Clinical Practice Guideline: Mechanical Ventilation in Adult Patients with Acute Respiratory Distress Syndrome. Am. J. Respir. Crit. Care Med..

[5] Butt Y., Kurdowska A., Allen T.C. (2016). Acute Lung Injury: A Clinical and Molecular Review. Arch. Pathol. Lab. Med..

[6] Mowery N.T., Terzian W.T.H., Nelson A.C. (2020). Acute lung injury. Curr. Probl. Surg..

[7] Long M.E., Mallampalli R.K., Horowitz J.C. (2022). Pathogenesis of pneumonia and acute lung injury. Clin. Sci..

[8] Xie M., Cheng B., Ding Y., Wang C., Chen J. (2019). Correlations of IL-17 and NF-κB gene polymorphisms with susceptibility and prognosis in acute respiratory distress syndrome in a chinese population. Biosci. Rep..

[9] Mikacenic C., Hansen E.E., Radella F., Gharib S.A., Stapleton R.D., Wurfel M.M. (2016). Interleukin-17A Is Associated with Alveolar Inflammation and Poor Outcomes in Acute Respiratory Distress Syndrome. Crit. Care Med..

[10] Yu Z.X., Ji M.S., Yan J., Cai Y., Liu J., Yang H.F., Li Y., Jin Z., Zheng J.-X. (2015). The ratio of Th17/Treg cells as a risk indicator in early acute respiratory distress syndrome. Crit. Care.

[11] Li M., Zhao Y., He J., Deng W., Cheng L., Jiang Z., Wang D. (2019). Protein Kinase C Theta Inhibition Attenuates Lipopolysaccharide-Induced Acute Lung Injury through Notch Signaling Pathway via Suppressing Th17 Cell Response in Mice. Inflammation.

[12] Li J.T., Melton A.C., Su G., Hamm D.E., LaFemina M., Howard J., Fang X., Bhat S., Huynh K.-M., O’Kane C.M. (2015). Unexpected Role for Adaptive αβTh17 Cells in Acute Respiratory Distress Syndrome. J. Immunol..

[13] Liu S.P., Huang L., Flores J., Ding Y., Li P., Peng J., Zuo G., Zhang J.H., Lu J., Tang J.-P. (2019). Secukinumab attenuates reactive astrogliosis via IL-17RA/(C/EBPβ)/SIRT1 pathway in a rat model of germinal matrix hemorrhage. CNS Neurosci. Ther..

[14] Mohamad H.E., Asker M.E., Shaheen M.A., Baraka N.M., Fantoukh O.I., Alqahtani A., Salama A.E., Mahmoud Y.K. (2023). Secukinumab and Black Garlic Downregulate OPG/RANK/RANKL Axis and Devitalize Myocardial Interstitial Fibrosis Induced by Sunitinib in Experimental Rats. Life.

[15] Liu S., Deng S., Ding Y., Flores J.J., Zhang X., Jia X., Hu X., Peng J., Zuo G., Zhang J.H. (2023). Secukinumab attenuates neuroinflammation and neurobehavior defect via PKCβ/ERK/NF-κB pathway in a rat model of GMH. Exp. Neurol..

[16] Oztanir M.N., Dogan M.F., Turkmen N.B., Taslidere A., Sahin Y., Ciftci O. (2022). Secukinumab Ameliorates Oxidative Damage Induced by Cerebral Ischemia-Reperfusion in Rats. Turk. Neurosurg..

[17] Karatas A., Celik C., Oz B., Akar Z.A., Etem E.O., Dagli A.F., Koca S.S. (2021). Secukinumab and metformin ameliorate dermal fibrosis by decreasing tissue interleukin-17 levels in bleomycin-induced dermal fibrosis. Int. J. Rheum. Dis..

[18] Vicovan A.G., Petrescu D.C., Cretu A., Ghiciuc C.M., Constantinescu D., Iftimi E., Strugariu G., Ancuta C.M., Caratașu C.-C., Solcan C. (2024). Targeting Common Inflammatory Mediators in Experimental Severe Asthma and Acute Lung Injury. Pharmaceuticals.

[19] Wang X., Zhang X., Sun L., Gao G., Li Y. (2022). Protective effect of Secukinumab on severe sepsis model rats by neutralizing IL-17A to inhibit IKBα/NFκB inflammatory signal pathway. Eur. J. Med. Res..

[20] Wang L., Wang X., Tong L., Wang J., Dou M., Ji S., Bi J., Chen C., Yang D., He H. (2018). Recovery from acute lung injury can be regulated via modulation of regulatory T cells and Th17 cells. Scand. J. Immunol..

[21] Righetti R.F., Santos TMd Camargo L.d.N., Aristóteles L.R.C.R.B., Fukuzaki S., Souza F.C.R.d., Santana F.P.R., de Agrela M.V.R., Cruz M.M., Alonso-Vale M.I.C., Tiberio I.D.F.L.C. (2018). Protective Effects of Anti-IL17 on Acute Lung Injury Induced by LPS in Mice. Front. Pharmacol..

[22] Camargo L.D.N., Righetti R.F., Aristóteles L.R.D.C.R.B., Dos Santos T.M., De Souza F.C.R., Fukuzaki S., Cruz M.M., Alonso-Vale M.I.C., Saraiva-Romanholo B.M., Prado C.M. (2018). Effects of Anti-IL-17 on Inflammation, Remodeling, and Oxidative Stress in an Experimental Model of Asthma Exacerbated by LPS. Front. Immunol..

[23] Camargo L.D.N., Santos T.M.D., Andrade F.C.P.D., Fukuzaki S., Dos Santos Lopes F.D.T.Q., De Arruda Martins M., Prado C.M., Leick E.A., Righetti R.F., Tibério I.D.F.L.C. (2020). Bronchial Vascular Remodeling Is Attenuated by Anti-IL-17 in Asthmatic Responses Exacerbated by LPS. Front. Pharmacol..

[24] Dos Santos T.M., Righetti R.F., Rezende B.G., Campos E.C., Camargo L.D.N., Saraiva-Romanholo B.M., Fukuzaki S., Prado C.M., Leick E.A., Martins M.A. (2020). Effect of anti-IL17 and/or Rho-kinase inhibitor treatments on vascular remodeling induced by chronic allergic pulmonary inflammation. Ther. Adv. Respir. Dis..

[25] Santos T.M.D., Righetti R.F., Camargo L.D.N., Saraiva-Romanholo B.M., Aristoteles L.R.C.R.B., De Souza F.C.R., Fukuzaki S., Alonso-Vale M.I.C., Cruz M.M., Prado C.M. (2018). Effect of Anti-IL17 Antibody Treatment Alone and in Combination With Rho-Kinase Inhibitor in a Murine Model of Asthma. Front. Physiol..

[26] Tsai Y.C., Cheng P.Y., Kung C.W., Peng Y.J., Ke T.H., Wang J.J., Yen M.-H. (2010). Beneficial effects of magnolol in a rodent model of endotoxin shock. Eur. J. Pharmacol..

[27] Lee Y.J., Lee Y.M., Lee C.K., Jung J.K., Han S.B., Hong J.T. (2011). Therapeutic applications of compounds in the *Magnolia* family. Pharmacol. Ther..

[28] Ni Y.F., Jiang T., Cheng Q.S., Gu Z.P., Zhu Y.F., Zhang Z.P., Wang J., Yan X.L., Wang W.P., Ke C.K. (2012). Protective effect of magnolol on lipopolysaccharide-induced acute lung injury in mice. Inflammation.

[29] Fu Y., Liu B., Feng X., Li F., Liang D., Liu Z., Li D., Cao Y., Zhang X., Zhang N. (2012). The effect of magnolol on the toll-like receptor 4/nuclear factor kappa B signaling pathway in lipopolysaccharide-induced acute lung injury in mice. Eur. J. Pharmacol..

[30] Lin M.H., Chen M.C., Chen T.H., Chang H.Y., Chou T.C. (2015). Magnolol ameliorates lipopolysaccharide-induced acute lung injury in rats through PPAR-γ-dependent inhibition of NF-kB activation. Int. Immunopharmacol..

[31] Tsai T., Kao C.Y., Chou C.L., Liu L.C., Chou T.C. (2016). Protective effect of magnolol-loaded polyketal microparticles on lipopolysaccharide-induced acute lung injury in rats. J. Microencapsul..

[32] Worthen G.S., Haslett C., Rees A.J., Gumbay R.S., Henson J.E., Henson P.M. (1987). Neutrophil-mediated pulmonary vascular injury. Synergistic effect of trace amounts of lipopolysaccharide and neutrophil stimuli on vascular permeability and neu-trophil sequestration in the lung. Am. Rev. Respir. Dis..

[33] Zhu C., Weng Q.Y., Zhou L.R., Cao C., Li F., Wu Y.F., Wu Y.-P., Li M., Hu Y., Shen J.-X. (2020). Homeostatic and early-recruited CD101− eosinophils suppress endotoxin-induced acute lung injury. Eur. Respir. J..

[34] Melgert B.N., Postma D.S., Kuipers I., Geerlings M., Luinge M.A., van der Strate B.W.A., Kerstjens H.A.M., Timens W., Hylkema M.N. (2005). Female mice are more susceptible to the development of allergic airway inflammation than male mice. Clin. Exp. Allergy.

[35] Debeuf N., Haspeslagh E., Van Helden M., Hammad H., Lambrecht B.N. (2016). Mouse Models of Asthma. CP Mouse Biol..

[36] Ehrentraut H., Weisheit C.K., Frede S., Hilbert T. (2019). Inducing Acute Lung Injury in Mice by Direct Intratracheal Lipopolysaccharide Instillation. JoVE.

[37] Huang Q., Han L., Lv R., Ling L. (2019). Magnolol exerts anti-asthmatic effects by regulating Janus kinase-signal transduction and activation of transcription and Notch signaling pathways and modulating Th1/Th2/Th17 cytokines in ovalbumin-sensitized asthmatic mice. Korean J. Physiol. Pharmacol..

[38] Gottlieb A.B., Deodhar A., Mcinnes I.B., Baraliakos X., Reich K., Schreiber S., Bao W., Marfo K., Richards H.B., Pricop L. (2022). Long-term Safety of Secukinumab Over Five Years in Patients with Moderate-to-severe Plaque Psoriasis, Psoriatic Arthritis and Ankylosing Spondylitis: Update on Integrated Pooled Clinical Trial and Post-marketing Surveillance Data. Acta Derm. Venereol..

[39] Percopo C.M., Brenner T.A., Ma M., Kraemer L.S., Hakeem R.M.A., Lee J.J., Rosenberg H.F. (2017). SiglecF+Gr1hi eosinophils are a distinct subpopulation within the lungs of allergen-challenged mice. J. Leukoc. Biol..

[40] Athari S., Nasab E., Athari S. (2018). Study effect of Ocimum basilicum seeds on mucus production and cytokine gene expression in allergic asthma mice model. Revue Française D’Allergologie.

[41] Tiotiu A., Badi Y., Kermani N.Z., Sanak M., Kolmert J., Wheelock C.E., Hansbro P.M., Dahlén S.-E., Sterk P.J., Djukanovic R. (2022). Association of Differential Mast Cell Activation with Granulocytic Inflammation in Severe Asthma. Am. J. Respir. Crit. Care Med..

[42] Wu L.C., Scheerens H. (2014). Targeting IgE production in mice and humans. Curr. Opin. Immunol..

[43] Van Der Pouw Kraan T.C.T.M., Van Der Zee J.S., Boeije L.C.M., DEGroot E.R., Stapel S.O., Aarden L.A. (1998). The role of IL-13 in IgE synthesis by allergic asthma patients. Clin. Exp. Immunol..

[44] Ho J.H.C., Hong C.Y. (2012). Cardiovascular protection of magnolol: Cell-type specificity and dose-related effects. J. Biomed. Sci..

[45] Wang J.P., Hsu E.F., Raung S.L., Chang L.C., Tsao L.T., Lin P.L., Chen C.C. (1999). Inhibition by Magnolol of Formylmethionyl-leucyl-phenyl alanine-induced Respiratory Burst in Rat Neutrophils. J. Pharm. Pharmacol..

[46] Scozzi D., Liao F., Krupnick A.S., Kreisel D., Gelman A.E. (2022). The role of neutrophil extracellular traps in acute lung injury. Front. Immunol..

[47] Li Y., Shen Y., Lin D., Zhang H., Wang T., Liu H., Wang Y. (2019). Neutrophils and IL17A mediate flagellar hook protein FlgE-induced mouse acute lung inflammation. Cell. Microbiol..

[48] Mosser D.M., Zhang X. (2008). Activation of murine macrophages. Curr. Protoc. Immunol..

[49] Abdelaziz M.H., Abdelwahab S.F., Wan J., Cai W., Huixuan W., Jianjun C., Kumar K.D., Vasudevan A., Sadek A., Su Z. (2020). Alternatively activated macrophages; a double-edged sword in allergic asthma. J. Transl. Med..

[50] Wang L., Wang D., Zhang T., Ma Y., Tong X., Fan H. (2023). The role of immunometabolism in macrophage polarization and its impact on acute lung injury/acute respiratory distress syndrome. Front. Immunol..

[51] Martín-Vicente P., López-Martínez C., Albaiceta G.M. (2022). The last-minute redemption of inflammatory cells in lung repair. Eur. Respir. J..

[52] Bouchery T., Harris N. (2019). The ins and outs of macrophages in tissue repair. Immunol. Cell Biol..

[53] Nguyen Q.T., Jang E., Le H.T., Kim S., Kim D., Dvorina N., Aronica M.A., Baldwin W.M., Asosingh K., Comhair S. (2019). IL-27 targets Foxp3^+^ Tregs to mediate antiinflammatory functions during experimental allergic airway inflammation. JCI Insight.

[54] Lu D., Lu J., Ji X., Ji Y., Zhang Z., Peng H., Sun F., Zhang C. (2020). IL-27 suppresses airway inflammation, hyperresponsiveness and remodeling via the STAT1 and STAT3 pathways in mice with allergic asthma. Int. J. Mol. Med..

[55] Kleinschek M.A., Owyang A.M., Joyce-Shaikh B., Langrish C.L., Chen Y., Gorman D.M., Blumenschein W.M., McClanahan T., Brombacher F., Hurst S.D. (2007). IL-25 regulates Th17 function in autoimmune inflammation. J. Exp. Med..

[56] Agrawal A., Zhuo H., Brady S., Levitt J., Steingrub J., Siegel M.D., Soto G., Peterson M.W., Chesnutt M.S., Matthay M.A. (2012). Pathogenetic and predictive value of biomarkers in patients with ALI and lower severity of illness: Results from two clinical trials. Am. J. Physiol.-Lung Cell. Mol. Physiol..

[57] Kong C.W., Tsai K., Chin J.H., Chan W.L., Hong C.Y. (2000). Magnolol attenuates peroxidative damage and improves survival of rats with sepsis. Shock..

[58] Wang W., Liang D., Song X., Wang T., Cao Y., Yang Z., Zhang N. (2015). Magnolol inhibits the inflammatory response in mouse mammary epithelial cells and a mouse mastitis model. Inflammation.

[59] Sun Y., Hu B., Stanley G., Harris Z.M., Gautam S., Homer R., Koff J.L., Rajagopalan G. (2023). IFN-γ Is Protective in Cytokine Release Syndrome-associated Extrapulmonary Acute Lung Injury. Am. J. Respir. Cell Mol. Biol..

[60] Strugariu G., Pomîrleanu C., Bran C., Costea A., Vicovan A., Tatarciuc D., Eșanu I., Ancuța E., Chirieac R., Ancuța C. (2022). The Prevalence of Atopy in Biologically Treated Spondyloarthropathies: A Retrospective Study of 200 Patients. J. Clin. Med..

[61] Lin S.P., Tsai S.Y., Lee Chao P.D., Chen Y.C., Hou Y.C. (2011). Pharmacokinetics, bioavailability, and tissue distribution of magnolol following single and repeated dosing of magnolol to rats. Planta Med..

[62] Jeong H.U., Kim J.H., Kong T.Y., Choi W.G., Lee H.S. (2016). Comparative metabolism of honokiol in mouse, rat, dog, monkey, and human hepatocytes. Arch. Pharm. Res..

[63] Zhu L., Ge G., Liu Y., He G., Liang S., Fang Z., Dong P., Cao Y., Yang L. (2012). Potent and selective inhibition of magnolol on catalytic activities of UGT1A7 and 1A9. Xenobiotica.

[64] Ghiciuc C.M., Vicovan A.G., Stafie C.S., Antoniu S.A., Postolache P. (2021). Marine-Derived Compounds for the Potential Treatment of Glucocorticoid Resistance in Severe Asthma. Mar. Drugs.

